# Supplementary material for: Multiple crossovers between positive and negative magnetoresistance versus field due to fragile spin structure in metallic GdPd3
Source: Sci Rep. 2017 Feb 17;7:42789. doi: 10.1038/srep42789 (PMC5314361; doi:10.1038/srep42789)
Supplement: Supplementary Information [file srep42789-s1.pdf]

Supplemental Material: Multiple crossovers between positive and negative magnetoresistance versus field due to fragile spin structure in metallic $GdPd_3$

Abhishek Pandey,[1,2,3] Chandan Mazumdar,[3] R. Ranganathan,[3] and D. C. Johnston[2]

[1]*Department of Physics and Astronomy, Louisiana State University, Baton Rouge, Louisiana 70803, USA*
[2]*Ames Laboratory-USDOE and Department of Physics and Astronomy, Iowa State University, Ames, Iowa 50011, USA*
[3]*Experimental Condensed Matter Physics Division, Saha Institute of Nuclear Physics, 1/AF, Bidhannagar, Kolkata 700064, India*

(A) Powder x-ray diffraction of $GdPd_3$:

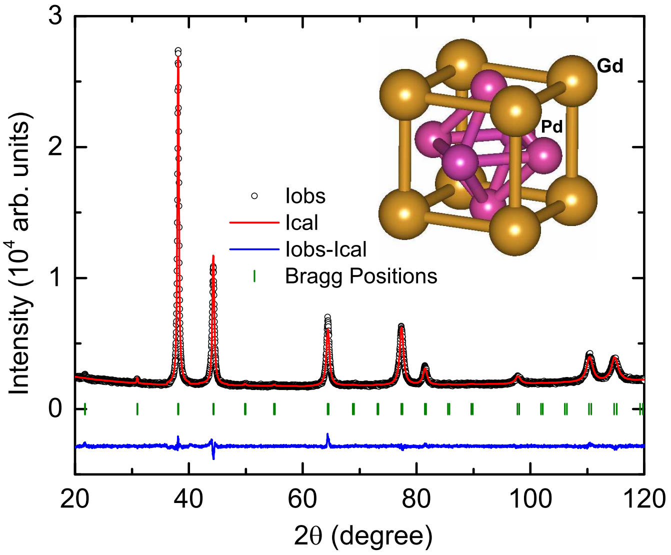

FIG. 1: Room temperature powder x-ray diffraction data, Rietveld refinement pattern, diffrence profile and the Bragg position for $GdPd_3$.The compound crystallizes in $AuCu_3$ type cubic structure with space group: $Pm\bar{3}m$ (No. 221). The refined value of the lattice lattice parameter a is 4.0919(4) Å. Inset: Crystal structure of $GdPd_3$.

(B) Electrical resistivity of $GdPd_3$:

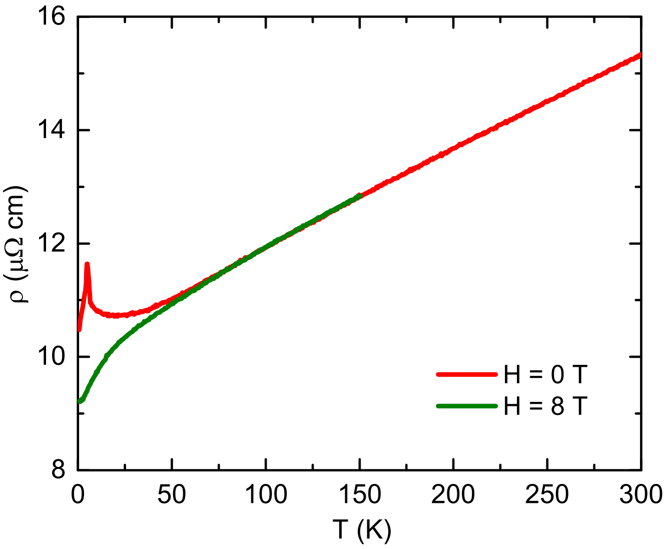

FIG. 2: Temperature dependence of resistivity ρ of $GdPd_3$ measured at two different applied magnetic fields $H = 0$ and 8 T.

(C) Heat Capacity of of YPd_3:

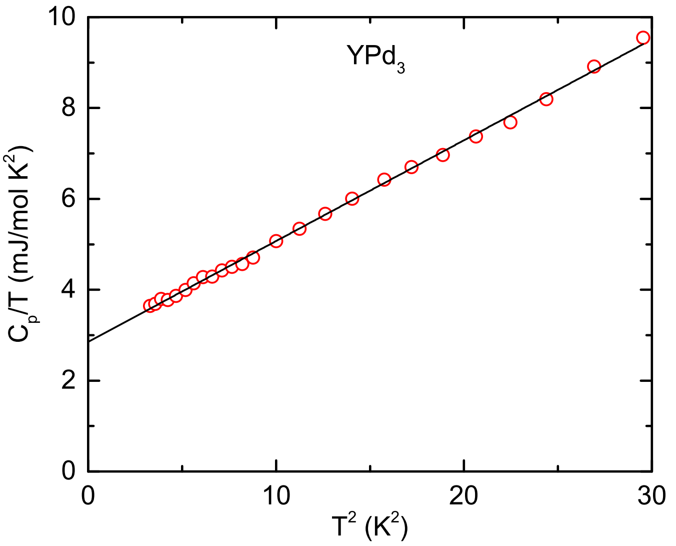

FIG. 3: Low temperature heat capacity C_p over T versus T^2 for YPd_3. The straight line is a fit by $C_p/T = \gamma + \beta T^2$ for $1.88\ \mathrm{K} \le T \le 5.43$ K, where $\gamma = 2.9(3)$ mJ/mol K^2 and $\beta = 2.22(2)$ J/mol K^4. From β we obtain the Debye temperature $\theta_D = 327(2)$ K.

(D) First derivative of magnetoresistance data at $T = 1.5$ and 2.0 K:

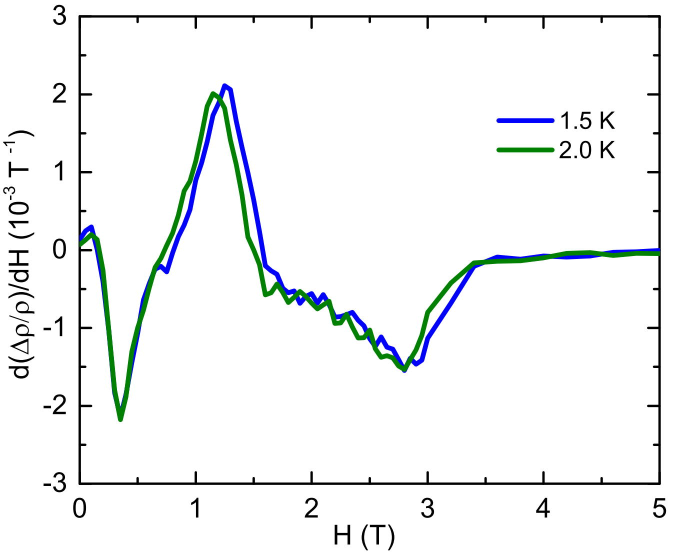

FIG. 4: First derivative of magnetoresistance data of $GdPd_3$ at two different temperatures $T = 1.5$ and 2.0 K.
